# Exploitation of High Tumour GSH Levels for Targeted siRNA Delivery in Rhabdomyosarcoma Cells

**DOI:** 10.3390/biom12081129

**Published:** 2022-08-17

**Authors:** Chengchen Duan, Helen Townley

**Affiliations:** 1 Nuffield Department of Women’s and Reproductive Health, Oxford University John Radcliffe Hospital, Headington, Oxford OX3 9DU, UK; 2 Department of Engineering Science, Oxford University, Parks Road, Oxford OX1 3PJ, UK

**Keywords:** CRYAB, HSPB2, KRT17, siRNA, rhabdomyosarcoma, near-infrared (NIR) imaging, glutathione

## Abstract

Metastatic alveolar rhabdomyosarcoma (aRMS) is an aggressive paediatric cancer with a poor prognosis. Downregulation of critical tumour genes using targeted siRNA remains an obstacle, but association with nanoparticles could help to deliver, protect, target, and enhance penetration. siRNA towards two genes was investigated: (i) Human αB-crystallin (CRYAB) and Heat Shock Protein Family B (Small) Member 2 (HSPB2), and (ii) Keratin 17 (KRT17). A mesoporous silica based nanosystem was linked to siRNA via disulfide bonds and loaded with IR820 dye. Transfection efficiency and signalling was evaluated, and the metabolic effects and cell proliferation were monitored in 2D culture and 3D spheroid models. The bound siRNA was protected from degradation with RNase I for at least 24 h. The delivered siRNA showed significant suppression of viability; 53.21 ± 23.40% for CRYAB and HSPB2 siRNA, and 88.06 ± 17.28% for KRT17 siRNA. After 72 h this increased to >50% cell apoptosis and necrosis. Intracellular total glutathione (GSH) levels were also compared with fibroblasts, and the RMS cell lines showed a several-fold increase. IR820 cellular uptake rate and penetration depth was significantly improved by nanoparticle delivery. Targetted siRNA delivery may pave the way for less invasive and more effective treatments of aRMS.

## 1. Introduction

As one of the most common and aggressive paediatric cancers, rhabdomyosarcoma (RMS) is believed to originate from skeletal muscle cells. There are two identified histological forms of RMS: embryonic RMS (eRMS) and alveolar RMS (aRMS) [1]. It has been shown that aRMS is more metastatic than the eRMS subtype, which normally leads to a poor prognosis [2]. Currently, most patients are treated with standard chemotherapy, radiotherapy, surgery, or combination therapy. These aggressive treatments normally lead to unavoidable life-long side effects, which is particularly poignant for paediatric patients. Combined with the lack of early diagnostic methods which leads to advanced metastasis, aRMS has a very low survival rate of less than 30% [3].

The discovery of siRNA technologies has been hailed as having great potential for improved oncology treatment. However, there are still many barriers blocking the clinical application of siRNA in vivo, such as nuclease degradation, non-specific binding with serum proteins, and kidney filtration [4,5]. In addition, the anionic charge of both the phosphate backbone and cell membranes increases the difficulty of passive diffusion of siRNA into cells [6]. Furthermore, bare siRNA has no targeting ability, which may lead to severe side effects in normal cells. Consequently, siRNA has only been used for limited clinical treatment, such as eyes or skin, which are externally accessed. Nanoparticles made of a variety of materials have been assessed for the protection of siRNA, including inorganic (e.g., silica [7], iron oxide [8], carbon [9]) and organic materials (e.g., liposomes [10]) [4,6,11]. Nanoparticles can passively accumulate in a tumour area via the enhanced permeability and retention (EPR) effect [12]. This couples fenestrations in the blood vessel endothelial cells in the tumour tissues with inadequate lymphatic drainage. After entering the tumour tissues, nanoparticles can assist in the passage of the associated siRNA across the cellular barrier [13]. This step is essential for the proper functioning of siRNA since electrostatic repulsion could prevent the negatively charged siRNA from crossing the cell membrane and accessing the cytoplasm [13]. Nanoparticle delivery of siRNA significantly decreases the possibility of being cleared by the immune system and reduces off-target effects compared with a traditional retroviral system or bare siRNA [4]. 

In this study, thiol functionalized mesoporous silica nanoparticles (SH-MSNPs) were chosen as the delivery core for the nanosystem. Silica nanoparticles have been widely used in various biomedical studies with well-established methods for controlling the size, shape, and morphology [14]. The siRNA was synthesized with a 5′ thiol functional group and formed a stable disulfide bond link with the SH-MSNP core. The bond is expected to remain stable until entering the high glutathione (GSH) environment of cancer cells [15]. GSH has been demonstrated as one of the special molecules which have specific biological roles in vivo. The reducing and detoxifying functionalities of GSH are enabled by one of the key functional groups with thiol group (-SH) from the cysteine residue. GSH is kept in the reduced form the majority of the time, before being transformed into the dimeric oxidised form GSH (GSSG) upon oxidative stress signalling. Thus, the GSH/GSSG ratio is a good indicator for the intracellular stress level; directly associated with cellular malfunction or multiple diseases. The GSH signalling molecule is also involved in metabolic activities, such as gene expression regulation, enzyme function, and protein synthesis. Intracellular glutathione (GSH) is more concentrated inside cells than extracellularly. Furthermore, the intracellular GSH concentration of cancerous cells is normally 1.7–7 fold higher than in normal cells [16,17]. High concentrations of GSH could efficiently break the disulfide bond linkage and allow targeted release within the cancer cells.

Two sets of targets for the effective gene knockdown in aRMS cells were assessed (i) Human αB-crystallin (CRYAB) and Heat Shock Protein Family B (Small) Member 2 (HSPB2), and (ii) Keratin 17 (KRT17). Both genes were found to be significantly upregulated in aRMS compared to eRMS or normal cells [18]. CRYAB and HSPB2 are closely linked and co-regulated by p53 [19]. Furthermore, CRYAB overexpression has been shown to be tightly associated with the migration and invasion of gastric cancer cells [20]. Previous research has shown that double knockout (DKO) of CRYAB and HSPB2 led to cell apoptosis and necrosis. This was due to a decrease in ATP production-related calcium-dependent respiration and metabolism, and a reduction in superoxide production [21]. KRT17, a type I keratin, was first found to relate to the growth of the human epithelium [22]. It has also been found to facilitate the development of cancers of the skin [23], colon and breast [24]. Knockdown of KRT17 could efficiently suppress cancer cell proliferation by inhibiting the AKT/mTOR/HIF1α pathway. 

The addition of near-infrared (NIR) dye to the nanoparticle system would enable the nanoparticles to be tracked both in vitro and in vivo [25] IR820 is a widely used NIR dye that allows high penetration depth NIR signalling but could also be potentially used for photothermal and photodynamic therapy [25]. Encapsulation also enhances the biocompatibility and stability of the IR820 dye.

Therefore, in this study, the GSH concentration of RMS cells has been measured as a fundamental property for the specific GSH-activated release. As such, the theranostic nanosystem is designed to protect the siRNA before specific release in the high GSH environment of aRMS cells, and to permit tracking with an NIR dye. 

## 2. Materials and Methods

### 2.1. Cell Lines and Tissue Culture 

Two different human rhabdomyosarcoma (RMS) cell lines acquired from the American Type Tissue Culture Collection (ATCC) (Manassas; VA, USA) were used in this study. RH30 (ATCC No. CRL-2061), which was selected to represent the alveolar RMS (aRMS), was acquired and derived from the bone marrow metastasis of a 17-year-old male. The other cell line selected to represent embryonal RMS (eRMS) was RD (ATCC no. CRL-7763), which was originally acquired and derived from a 7-year-old female’s muscle carcinoma. Human primary fibroblasts were selected as the normal control cell line. The fibroblast cell line was kindly provided by Dr Jo Poulton from the Nuffield Department of Women’s & Reproductive Health, University of Oxford.

Cells were incubated in a humidified 5% CO_2_ incubator at 37 °C with high-glucose Dulbecco’s Modified Eagle’s Medium (DMEM) (Sigma-Aldrich, Poole, UK). DMEM culture medium was pre-added with 2 mM l-glutamine (Gibco, Life Technologies Ltd., Renfrew, UK), penicillin (100 U/mL) and streptomycin (100 g/mL) (Gibco, Life Technologies Ltd., Renfrew, UK) and 10% (*v/v*) Foetal Bovine Serum (FBS) (Sigma-Aldrich, Poole, UK). T75 Tissue Culture Treated Flasks (Nunc, Thermofisher Scientific Renfrew, Renfrew, UK) were used for the day-to-day culture. Trypsin (0.25% *w/v*) containing EDTA (0.02% *w/v*) was used to detach cells from the bottom of flasks (Sigma-Aldrich, Poole, UK).

### 2.2. Cell Viability and Metabolic Assay (MTT)

A colourimetric MTT assay (3-(4,5-Dimethylthiazol-2-yl)-2,5-Diphenyltetrazolium Bromide) (Sigma- Aldrich, Poole, UK) was used to assess cell proliferation and metabolic levels. Cells were seeded at a density of 1 × 10^4^ cells per well in a 96-well microplate and incubated for 24 h for full attachment to the bottom of wells. 

The cells were treated with one of the following: (i) buffer alone, (ii) free thiol functionalized siRNA, (iii) SH-MSNPs, (iv) siRNA with DharmaFect transfection reagent (Dharmacon, Lafayette, CO, USA), or (v) purified siRNA linked mesoporous silica nanoparticles (MSNPs). The aliquoted siRNA stock solution (20 μM in 1x siRNA buffer (60 mM KCl, 6 mM HEPES-pH 7.5, 0.2 mM MgCl_2_)) (Horizon Discovery, Cambridge, UK) was prepared and applied for transfection. For DharmaFect induced transfection, the reaction was prepared to start from two tubes: tube 1 containing siRNA and serum-free DMEM mixed at a ratio of 1:19, and tube 2 containing DharmaFect and serum-free DMEM mixed at a ratio of 1:49. Enough solution was prepared for at least 100 μL for each well. After 5 min, the two tubes were thoroughly mixed and incubated for 20 min at room temperature. Before transfection, the cells were washed once with PBS and an antibiotic-free medium. 

After selected cell lines were treated with set concentrations of siRNA or siRNA linked MSNPs for a defined period of time, the medium was discarded from the microplate well after treatment. Subsequently, MTT medium solution (100 mL of growth media with 0.5 mg/mL of MTT) was added into each well followed by 3 h incubation at 37 °C to allow the formation of purple formazan crystals within the cells. The formation of crystals was then validated before moving on to the next stage. All supernatant was carefully discarded and 100 µL dimethyl sulfoxide (DMSO) (Sigma- Aldrich, Poole, UK) was added into each well to solubilize the formazan. The microplate was incubated for 20 min and then shaken for 2 min before measurement. The absorbance was read at 575 nm using the plate reader following incubation (Tecan Infinite^®^ 200 PRO, Reading, UK).

### 2.3. Scratch Assay

A scratch test was performed to assess cell migration and invasion. RMS cells were cultivated in a 12-well sterile microplate at a density of 3 × 10^5^ per well. Cells were allowed to grow, spread, and form a confluent monolayer for 48 h. A 1 mL pipette tip was used as a pin tool to scratch through the cell layer and remove the scratched cells, resulting in the formation of a cross-like cell-free zone in each well with a constant width.

After incubation with different concentrations of siRNA or siRNA linked MSNPs, cells would migrate inward to the scratched area at a different speed. For comparison of cell mobilities, images of scratches were taken before and after the incubation for different wells. The average width of the scratched cross was measured twice across the horizontal and vertical scratches near the centre of the cross. The cell mobilities were quantified using the difference in scratch width before and after incubation. 

### 2.4. Synthesis of Thiol Functionalized MSNPs (SH-MSNPs)

The SH-MSNP synthesis method was modified and optimized from a published protocol [26]. Hexadecyltrimethylammonium Chloride (2 g; CTAC) (Sigma-Aldrich, Poole, UK) and Triethylamine (0.06 g; TEA) (Sigma-Aldrich, Poole, UK) were dissolved and added into 20 mL of 95 °C distilled water under intensive stirring. After 1 h of stirring Tetraethyl orthosilicate (1.5 mL; TEOS) (Sigma-Aldrich, Poole, UK) was added dropwise into the mixture. After a further 1 h, 200 μL of a 1:1 mixture of TEOS and 3-mercaptopropyl triethoxysilane (MPTES) (Sigma-Aldrich, Poole, UK) was added into the reaction. The synthesized nanoparticles were collected by high-speed centrifugation and washed with 99% ethanol three times. The obtained SH-MSNPs were vacuum dried and stored at room temperature. 

### 2.5. Nanoparticles Characterization

For the preparation of specimens for transmission electron microscopy (TEM), the synthesized SH-MSNPs were dispersed in ethanol and dropped onto holey carbon-coated TEM grids (Agar Scientific, Stansted, UK) until fully dried. A JEOL-2100 TEM (JEOL, Tokyo, Japan) was used for TEM imaging, operating at 200 kV. Gatan 3 microscopy suite software (Gatan, Inc., Pleasanton, CA, USA) was used for TEM image analysis. 

Dynamic Light Scattering (DLS) and particle surface ζ (zeta) potential was measured with a Zetasizer Nano ZS90 (Malvern Panalytical, Malvern UK). The SH-MSNPs were suspended in 1 × PBS buffer (pH = 7.4) prior to the measurement to quantify the surface charge for particles in a colloidal suspension. For calculating electrophoretic mobility, zeta potential, and zeta potential distribution, 100 runs were read for every sample.

Thermogravimetric analysis (TGA) (Perkin Elmer TGA7, USA) was used to analyse nanoparticles from 110 °C to 800 °C at a rate of 10 °C per minute under a nitrogen atmosphere. 

For validation of surface thiol bonds, the Raman spectra of SH-MSNPs were read using a confocal Horiba Jobin-Yvon Raman microscope (Horiba, Glasgow, UK) equipped with a 532-nm neodymium–yttrium aluminium garnet laser. The laser power on samples was 12 mW after attenuation by neutral density filters.

### 2.6. siRNA Linkage with SH-MSNPs and Validation of Binding

Thiol functionalized siRNA was custom synthesized by Merck (Merck, Haverhill, UK).

i. The sequences of the siRNA targeted to CRYAB mRNA were:

5′-[ThiC6]CUGUGAAUGGACCAAGGAA[dT][dT]-3′ (sense strand) 

and 5′-[ThiC6]UUCCUUGGUCCAUUCACAG[dT][dT]-3′ (antisense strand).

ii. The sequence of the siRNA targeted to HSPB2 mRNA was:

5′-[ThiC6]CUCCCAUGAUGGCAUCUUA[dT][dT]-3′ (sense strand) 

and 5′-[ThiC6]UAAGAUGCCAUCAUGGGAG[dT][dT]-3′ (antisense strand).

iii. The sequence of the siRNA targeted to KRT17 mRNA was:

5′- [ThiC6]CCAGUACUACAGGACAAUU[dT][dT]-3′ (sense strand) 

and 5′-[ThiC6]AAUUGUCCUGUAGUACUGG[dT][dT]-3′ (antisense strand). 

siRNA was re-suspended in 1x siRNA buffer which contained 30 mM HEPES-pH 7.4, 300 mM KCl, 1.0 mM MgCl_2_ and RNase-free water (Dharmacon, Lafayette, CO, USA). The solubilized siRNA samples were then aliquoted into several tubes containing 50 µL of 20 µM siRNA stock suspension within the RNase free environment. 

Prior to the linkage, all 5′-thiol functionalized siRNA were obtained in a disulfide form for stability. In order to reduce the disulfide bond of the thiol-modified 5′ end to an active sulfhydryl form, 100 mM dithiothreitol (DTT) solution was added to 100 mM sodium phosphate buffer (pH 8.3–8.5). Aliquoted siRNA stock solution (50 µL) was mixed with 125 µL of the DTT solution, followed by incubation for 1 h at room temperature. The by-product was carefully removed using a NAP-10 column and 100 mM sodium phosphate buffer (pH 6.0) following the manufacturer’s protocol (GE Healthcare, Illinois, USA). The final activated product was eluted with 200 µL of sodium phosphate buffer (pH 6.0). All buffer was prepared with RNase-free water. The concentration of activated siRNA was measured with NanoQuant Plate™ using Infinite^®^ 200 PRO plate reader dsRNA reading function (Tecan, Reading, UK). 

Immediately after the activation and concentration reading, thiol functionalised siRNA (CRYAB and HSPB2, or KRT17) was mixed with 1 mg of SH-MSNPs dispersed in 1 mL of sodium phosphate buffer (pH = 7.4). The binding reaction was kept for 8 h within an RNase free environment at room temperature. siRNA linked MSNPs were collected by centrifugation at 15,000 rpm for 30 min. The same amount of RNase free buffer was then added for re-dispersion of collected siRNA linked MSNPs. The re-dispersion and centrifugation process was repeated four times, and all supernatant were collected for the reading of remaining free siRNA with NanoQuant Plate™. 

For validation of siRNA linkage with SH-MSNPs, the purified siRNA linked MSNPs and free siRNA samples were run on a 2% (*w/v*) of native agarose gel electrophoresis for 1 h at 100 V. The gel was prepared in 0.5× TBE buffer supplemented with 0.005% (*v/v*) EtBr and 10 mM MgCl_2_.

### 2.7. Validation of RNase I Protection

For validation of the protective effect of MSNPs to the linked siRNA, the siRNA linked MSNPs or free siRNA targeting different mRNAs were incubated separately with 0.25% (*w/v*) RNase I (Sigma-Aldrich, Poole, UK) for different periods of time, ranging from 15 min to 4 h (for free siRNA) or even longer, up to 24 h. Gel electrophoresis was used to assess degradation as above. As previously, 2% (*w/v*) native agarose gel electrophoresis was used to assess degradation for 1 h at 100 V. The gel was prepared under the same conditions. 

### 2.8. Allophycocyanin(APC) Annexin V/ Propidium Iodide (PI) Assay 

Pure SH-MSNPs or siRNA-MSNPs treated RH30 spheroids were collected and dissociated into single cells by trypsin treatment and centrifugation. The cells were then washed three times with 200 μL of cold 1 × PBS and Annexin V binding buffer (BioLegend, CA, USA). The washed cells were then incubated with 50 μL of Annexin V binding buffer which was pre-mixed with 2.5 μL of PI and 5 μL of APC Annexin V (BioLegend, CA, USA) at room temperature for 15 min. The cells were then collected by centrifugation and resuspended in 400 μL of Annexin V binding buffer for flow cytometry evaluation. Flow cytometry was performed using a BD FACS Calibur (BD, NJ, USA) and evaluated in both the FL3-H and FL4-H channels. 

### 2.9. IR820 NPs Loading in siRNA Linked MSNPs 

To load NIR dye IR820 (Sigma-Aldrich, Poole, UK) into siRNA linked MSNPs, 200 μL of IR820 stock solution (84.90 mg/mL) was prepared by dissolving IR820 in DMSO. The stock solution was then diluted 1000 times with RNase free water. Diluted IR820 solution (4 mL) was then mixed with siRNA linked MSNPs with shaking at room temperature for 24 h in a dark environment. The particles were washed with Rnase free water and collected by ultrahigh-speed centrifugation (20,000 rpm, 10 min); this was repeated several times. The final product (IR820 loaded siRNA-MSNPs) was stored in Rnase free PBS buffer at 4 °C.

### 2.10. Flow Cytometry for IR820 Delivery Evaluation

In order to validate the IR820 NIR signal penetration rate, RH30 sarcoma spheroids were prepared before incubation for 24 h with either (i) PBS, (ii) the same concentration of free IR820 NIR dye, or (iii) IR820 loaded siRNA linked MSNPs. 

Likewise, for comparing the penetration speed between free IR820 and IR820 loaded siRNA linked MSNPs in a time-wise manner, RH30 sarcoma spheroids were prepared and incubated with the same concentration of either free IR820, or IR820 loaded siRNA linked MSNPs for different periods of times, ranging from 0~24 h. 

Similar to the apoptosis/necrosis assay, the treated RH30 spheroids were collected and dissociated into single cells by trypsin treatment and centrifugation. The cells were then washed three times with 500 μL of cold 1xPBS to remove any non-specific binding dye or particles. All the collected cells were fixed with a 4% (*v/v*) PFA solution (Bio-rad, Watford, UK) prior to the next step of flow cytometry analysis. Flow cytometry was performed using a BD FACS Calibur and evaluated in the FL4-H channel. The amount of IR820 signal was quantified by normalized median fluorescence intensity. The entire procedure was carried out on ice in darkness. The experiment was repeated in duplicate on three separate occasions.

### 2.11. Confocal Imaging of Efficient IR820 Cell Penetration Delivered with MSNPs

Cells were seeded at a density of 1 × 10^5^ cells per well in complete DMEM medium which was pre-added with 2 mM l-glutamine (Gibco, Life Technologies Ltd., Renfrew, UK), penicillin (100 U/mL) and streptomycin (100 g/mL) (Gibco, Life Technologies Ltd., Renfrew, UK) and 10% (*v/v*) Foetal Bovine Serum (FBS) (Sigma-Aldrich, Poole, UK), in glass-bottom imaging petri dishes (ibidi GmbH,, Gräfelfing, Germany) and allowed to adhere to the surface for 24 h in a 37 °C tissue culture incubator. RH30 sarcoma cells were incubated overnight with the same concentration of either free IR820 or IR820 loaded siRNA linked MSNPs, respectively. The cells were washed three times with cold PBS after incubation to remove any non-specific binding. The entire procedure was carried out in complete darkness. Before imaging, the cells were maintained in warm PBS and stained for 15 min with 0.1 g/mL DAPI (Thermofisher Scientific, Renfrew, UK) and 1:200 diluted CellBrite^TM^ Green Cytoplasmic Membrane Dye (Biotium, CA, USA). All samples were then fixed with a 4% (*v/v*) PFA solution. A Leica SP8 confocal microscope was used for the imaging (Leica Biosystems, Nußloch, Germany) 

### 2.12. 3D Spheroid Generation and Growth with Treatment

The 3D RMS spheroids were grown on 1% (*w/v*) agarose-coated 96-well plates. To prepare the plate, powder-form low electroendoosmotic (EEO) agarose (Sigma-Aldrich, Poole, UK) was fully dissolved in 1 × PBS with very mild heating until the formation of a clear solution (Sigma- Aldrich, Poole, UK). Warm agarose solution (100 μL) was carefully added a the flat-bottom 96-well plate, ensuring that no bubbles were present. The plate was left to dry and sterilized under UV for 2 h at room temperature. A flat solid agarose layer could be seen to form in the well after this step. RMS cells from cultures were washed with DMEM medium and seeded at a density of 100,000 cells per well onto the agarose-coated plate without damaging the agarose layer. The plate was then cultured at 37 °C with 5% CO_2_ in a tissue culture incubator for 4 days. The growth of the spheroids was monitored every day, and after 4 days they were transferred into a normal 96-well plate for experiments. 

The RMS spheroids were incubated for 72 h with either (i) PBS, (ii) the same concentration of free IR820 NIR dye, or (iii) IR820 loaded siRNA linked MSNPs. The sizes of the spheroids were monitored at *t* = 0 and every 24 h afterwards using a Motic 101M microscope with a Moticam 2500 camera and MHG 100B laser (Motic, Xiamen, China). The diameters of spheroids were quantified using ImageJ (NIH, Bethesda, USA).

### 2.13. Measurement of Intracellular GSH/GSSG Level

A Promega GSH/GSSG-Glo^TM^ Assay was used to quantify the intracellular concentration of total GSH and GSSG (Promega, Madison, WI, USA). In 96-well plates, normal cells (fibroblasts) and RMS cells were plated at a concentration of 1 × 10^4^ cells per well. The cells were allowed to adhere to the wells overnight, and the assay was carried out according to the manufacturer’s instructions. The medium was then discarded, and the cells were collected accordingly. The collected cells were then treated with either total glutathione lysis reagent or oxidised glutathione lysis reagent within the kit (Promega, Madison, WI, USA). Both plates were maintained at room temperature for 30 min before adding the luciferin production reagent. The plates were briefly shaken inside the plate reader (Tecan Infinite^®^ 200 PRO, Reading, UK) before being allowed to equilibrate for 15 min at room temperature. 

The Luminescence was then measured using a plate reader luminescence function (Tecan Infinite^®^ 200 PRO, Reading, UK), and the GSH and GSSG amount was calculated according to the standard curve generated by the standard samples tested in plate at the same time. The following equation was used to determine GSH/GSSG ratios:

GSH/GSSG = [Total GSH − (2 GSSG)]/GSSG.

### 2.14. Statistics and Reproducibility

All statistical analyses of experimental data were performed using Microsoft™ Excel (Microsoft, Redmond, WA, USA). GraphPad Prism 8.0.2 (GraphPad Prism, La Jolla, CA, USA) was used for graph plotting. Error bars in the experiments indicate standard deviation (SD). The difference was considered significant and shown when the p values were less than 0.05. The figure legends include information on the number of events and independent experiments, as well as statistical data and methodologies. 

### 2.15. Other Software

FlowJo VX (FlowJo, LLC, OR, US) was used to analyse and compare flow cytometry data. For all confocal image analysis and measurement, the Leica Application Suite X (LAS X) (Leica Biosystems, Nußloch, Germany) was employed.

## 3. Results

### 3.1. Evaluation of Selected siRNAs

**Figure 1:**
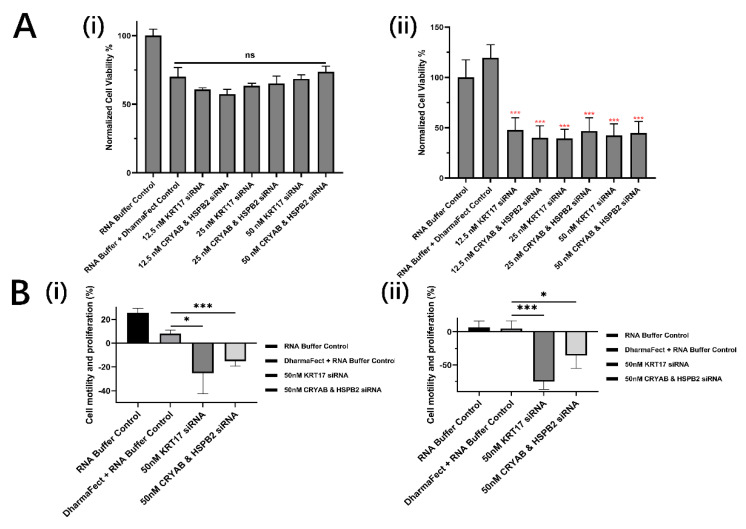
Measurement of chosen siRNAs’ effects on cell proliferation and mobilities. (**A**) MTT results comparing the cell relative survival rate and metabolism level of RD (i) and RH30 (ii) sarcoma cells after treated with different concentrations of KRT17-targeting siRNA, or CRYAB and HSPB2-targeting siRNA using DharmaFect as transfection reagent for 24 h (*n* = 6). (**B**) Scratch assay results comparing the cell relative mobility of RD (i) and RH30 (ii) sarcoma cells after treated with 50 nM of KRT17-targeting siRNA, or CRYAB and HSPB2-targeting siRNA using DharmaFect as transfection reagent for 24 h. Data are presented as mean ± SD for each individual cell line (*n* = 3). Significance was tested using a two tailed t-test compared to the cells with only buffers for each cell line (ns: not significant, * *p* ≤ 0.05, *** *p* ≤ 0.001).

The genes CRYAB and HSPB2, and KRT17, were investigated as candidates for knockdown in aRMS cells. RH30 cells (aRMS) were used for investigations and compared with RD cells (eRMS). Both cell lines were transfected with CRYAB and HSPB2, or KRT17 siRNA, ranging from 12.5 nM to 50 nM. The eRMS cell line showed no significant reduction in proliferation rate compared with the control (Figure 1A(i)). Conversely, the cell viability and metabolism of the aRMS cell line (RH30) were both significantly suppressed. Cell growth was decreased to lower than 50%, 24 h after transfection (Figure 1A(ii)). For the CRYAB and HSPB2 DKO, the cell viability of RH30 cells was suppressed to 39.92%, 46.68%, and 44.68% for 12.5 nM, 25 nM, and 50 nM of both siRNAs, respectively. Similarly, after the KRT17 knockout, the cell viability of RH30 cells decreased to 47.74%, 39.31%, and 42.35% for 12.5 nM, 25 nM, and 50 nM of siRNA, respectively. For the three concentrations applied, although they all showed significant suppression compared with both cell control and the buffer control, there was no significant difference with increasing concentration or the siRNA groups, with viabilities all around 45%.

While cell viability was only affected in RH30 cells after the siRNA transfection for both CRYAB and HSPB2 DKO and KRT17 knockout, the cell invasion, as determined by the scratch test, showed significant inhibition in both RD and RH30 cells, albeit to different degrees. After transfection with 50 nM siRNA, RD cells showed a decrease in mobility of 25.17% with KRT17 siRNA, and 15.08% after with CRYAB and HSPB2 (Figure 1B(i)). However, much greater inhibition was seen in RH30 cells, with 74.95% decrease with KRT17 siRNA and 35.79% decrease with CRYAB and HSPB2 siRNA (Figure 1B(ii)). 

### 3.2. Thiol Functionalized Mesoporous Silica Nanoparticles (SH-MSNPs)

**Figure 2:**
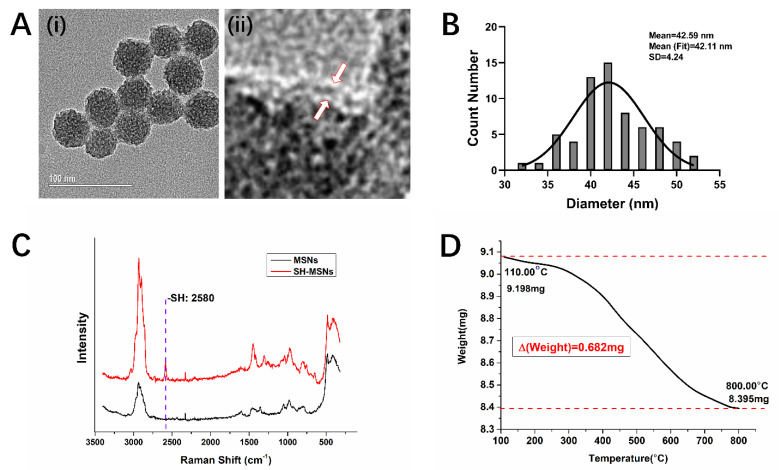
Characterization of synthesized SH-MSNPs and thiol bonds on the particle surface. (**A**) TEM images of (i) synthesized 50 nm SH-MSNPs, (ii) enlarged surface thiol bond layer of SH-MSNPs (layer indicated by arrows). (**B**) Gaussian distribution of SH-MSNPs diameter measured from the TEM images. (**C**) Thiol bond was detected using Raman Spectroscopy. A unique 2580 cm^−1^ peak was shown in SH-MSNPs, but not in MSNPs. (**D**) The amount of functionalized thiol bond was quantified using TGA from 110 °C to 800 °C in nitrogen environment.

Nanoparticles based on mesoporous silica were synthesized as the core of the system. The surface of the nanoparticles was functionalized with thiol bonds for attachment and targeted release of the siRNA [26]. The diameter of these SH-MSNPs was monitored by multiple methods. The hydrodynamic diameter was determined to be 73.57 ± 17.12 nm by dynamic light scattering (DLS; Supplementary Figure S1). TEM images of the SH-MSNPs indicated a spherical shape with a mesoporous surface. The diameters of irregular mesopores on the surface appeared to be around 1–2 nm by random measurement of 100 mesopores from the TEM image (Figure 2A(i)). Furthermore, a very thin 1 nm surface layer was observed on the SH-MSNPs which could be considered as supplementary evidence of a thiol bond layer (Figure 2A(ii)). The average diameter was calculated as 42.59 ± 4.24 nm through randomly counting 100 nanoparticles from images. All measured diameters were grouped and Gaussian fitted, and all particle diameters were well distributed in a very narrow range and the mean value was 42.11 ± 4.12 nm. This showed a uniform distribution of particle diameter (Figure 2B). In a neutral pH, the surface potential of SH-MSNPs was determined to be −23.3 ± 7.50 mV (Supplementary Figure S2). The strong surface potential could keep the nanoparticles moderately stable in an aqueous environment.

Raman Spectroscopy was used to compare the synthesized SH-MSNPs with bare MSNPs for further validation of the surface thiol bonds. SH-MSNPs showed a very strong peak at 2580 cm^−1^ which was absent from bare MSNPs. This peak is a fingerprint peak for the thiol group [27]. Moreover, the thiol bond was linked to the silica network through –CH_2_-CH_2_-CH_2_-SH. Raman fingerprints of —C at 1305 cm^−1^ and C—S at 653 cm^−1^ were also observed from the comparison (Figure 2C) [28].

Subsequently, the surface thiol groups were quantified by Thermal Gravimetric Analysis (TGA). From the weight decrease, the amount of thiol groups on the surface was calculated as 0.065 mol per 1 mol SH-MSNPs. This amount would dictate the amount of siRNA the particle could carry (Figure 2D).

### 3.3. Linkage and Protection of siRNAs on the SH-MSNPs Surface

**Figure 3:**
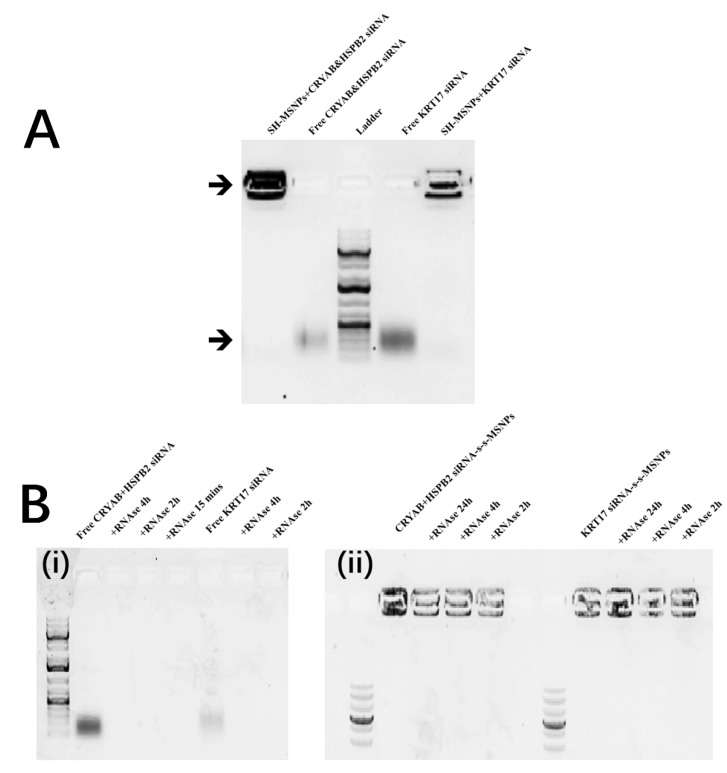
Electrophoresis validation of siRNA linkage and protection by SH-MSNPs (**A**) Validation of siRNA linkage onto the surface of SH-MSNPs electrophoresis gel image of both free CRYAB and HSPB2-targeting siRNA, or KRT17-targeting siRNA and siRNA linked SH-MSNPs for the validation of disulfide bond linkage, cropped gel image. (**B**) Evaluation of siRNA protection after linked onto the surface of SH-MSNPs electrophoresis gel image of (i) free CRYAB and HSPB2-targeting siRNA, or KRT17-targeting siRNA and (ii) siRNA linked SH-MSNPs, treated with same concentration of RNase for different periods of time at 37 °C for the evaluation of degradation prevention after linked with SH-MSNPs, cropped gel image.

The siRNAs were linked to the surface of the MSNPs via disulfide bonds linkages. To evaluate the binding amount and capacity, free siRNAs left in the incubation buffer were collected and quantified. The effective binding ratios were calculated, and it was shown that siRNA was bound to the MSNPs surface at similar levels; 51.41% and 49.46% for the linkage of CRYAB and HSPB2 siRNA, and KRT17 siRNA, respectively. In order to further validate the stable linkage between siRNA and MSNPs, both the CRYAB and HSPB2 and KRT17 siRNA linked MSNPs were examined by 1% agarose gel. While free siRNA (or ladder) could move freely within the agarose gel, the stable linkage with MSNPs prevented any electrophoretic migration, i.e., fluorescent signal remained in the wells. No visible amounts of siRNA release could be detected by electrophoresis from the siRNA linked MSNPs, demonstrating that the siRNA was strongly attached to the surface of the MSNPs via disulfide bonds (Figure 3A).

In addition, samples were incubated with RNase I, an enzyme which could easily degrade unbound RNA. As shown in Figure 3B(i), 5 nM of free siRNA was cleared by the RNase I in 15 min (irrespective of the sequence). On the contrary, the MSNPs were seen to protect the bound siRNAs from degradation for significantly longer time, with a substantial amount of siRNA visualised after electrophoresis in all the RNase I challenge groups after 2 h, 4 h, or 24 h of incubation (Figure 3B(ii)). 

### 3.4. siRNA Linked MSNPs Effects on Cell Proliferation and Mobilities

**Figure 4:**
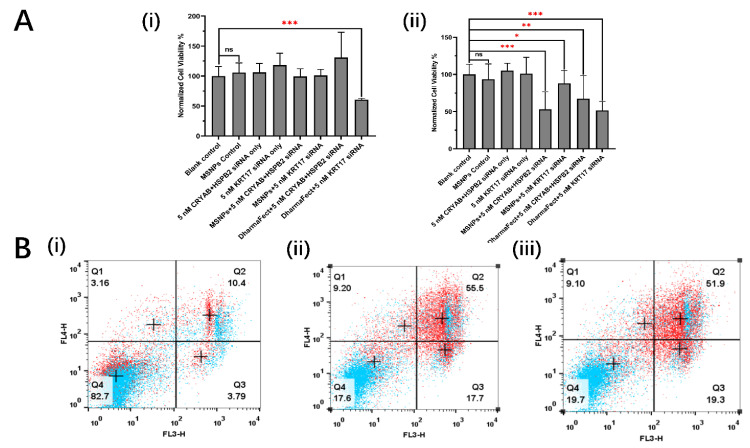
Measurement of siRNA linked MSNPs effects on cell proliferation and mobilities (**A**) MTT results comparing the cell relative survival rate and metabolism level of RD (i) and RH30 (ii) sarcoma cells after treatment with 5 nM of free CRYAB and HSPB2-targeting siRNA, or KRT17-targeting siRNA, or the same concentration transfected by DharmaFect or MSNPs for 24 h (*n* = 6). Data are presented as mean ± SD for each individual cell line (*n* = 3). Significance was tested using a two tailed t-test compared to the cells with only buffers for each cell line (ns: not significant, * *p* ≤ 0.05, ** *p* ≤ 0.01, *** *p* ≤ 0.001). (**B**) APC-Annexin V and PI assay results measuring the cell apoptosis and necrosis RH30 sarcoma cell spheroids with SH-MSNPs control (i), 50 nM of CRYAB and HSPB2-target siRNA linked MSNPs (ii), and 50 nM of KRT17-target siRNA linked MSNPs (iii) for 72 h. Control groups: blue; treatment groups: red.

The effects on aRMS cell proliferation and metabolism after incubation with siRNA linked MSNPs were evaluated in comparison with the standard lipid transfection reagent DharmaFect™. After 24 h of incubation, it was found that the carrier only (SH-MSNPs) control did not affect either RD or RH30 cells. Similarly, free siRNAs did not affect either RD or RH30 cells with regards to proliferation or metabolic rate. However, when it came to the transfection with the help of DharmaFect or MSNPs carriers, RD and RH30 reacted differently with the same concentration of siRNAs. After siRNA transfection with DharmaFect, the RD cells did not show much difference in the viability level compared with the control group, except for the 5 nM KRT17 siRNA knockout group. The viability decreased to 60.75 ± 1.79% after 24 h of transfection (Figure 4A(i)). Nevertheless, RH30 cells, which had a much higher expression for all targeted mRNA, were all very sensitive with the transfection. Through the transfection of 5 nM siRNA with DharmaFect, the viability of RH30 cells was significantly decreased to 67.16 ± 37.17% for CRYAB and HSPB2 DKO, and 51.64 ± 12.04% for KRT17 knockout (Figure 4A(ii)). 

Similar or better outcomes were shown in the MSNPs transfection groups. With the same amount of transfected siRNA, the viability of RH30 cells was significantly decreased to 53.21 ± 23.40% for CRYAB and HSPB2 DKO, and 88.06 ± 17.28% for KRT17 knockout (Figure 4A(ii)). 

In addition to 2D culture, the effects of knockdown were also examined in 3D spheroid models. After incubation with 50 nM KRT17, or CRYAB and HSPB2 siRNA- MSNPs, an APC-Annexin V/PI assay was used to evaluate cell apoptosis and necrosis after 72 h. After incubation of RH30 cell spheroids with SH-MSNPs carrier control only 3.16% and 10.4% of cells were found to be in the early/ late apoptosis and necrosis stage, respectively (Figure 4B(i)). However, SH-MSNPs conjugated with CRYAB and HSPB2 showed 9.20% of cells in early/late apoptosis, and 55.5% of cells in the necrosis stage, respectively (Figure 4B(ii)). Similar results were seen after KRT17 knockdown; 9.10% in early/late apoptosis and 51.9% in necrosis (Figure 4B(iii)). 

### 3.5. Cellular Uptake and Effects on Cell Proliferation of IR820 Loaded siRNA linked MSNPs 

**Figure 5:**
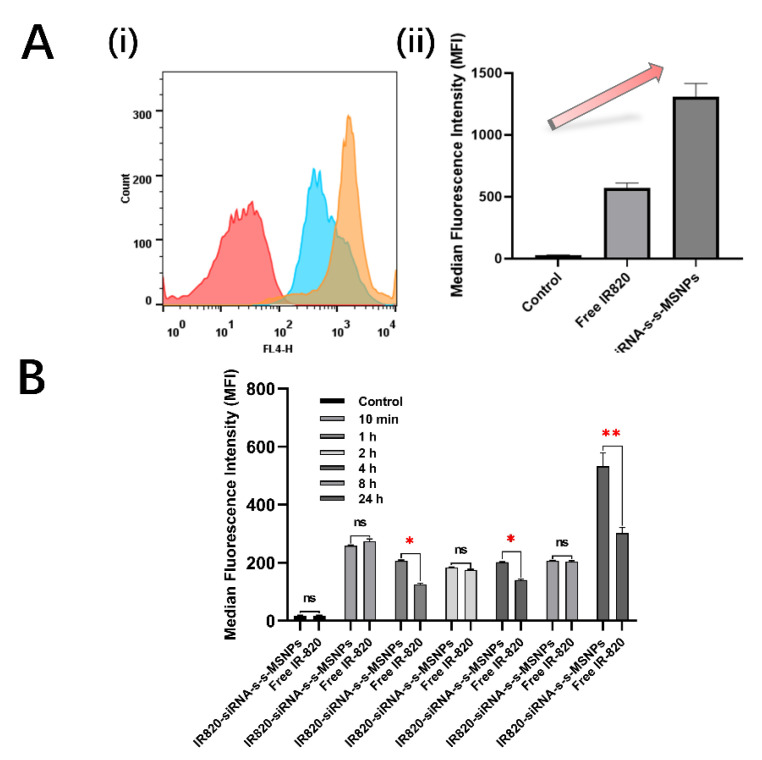
Evaluation of IR820 NIR signal penetration rate in RH30 sarcoma spheroids measured by flow cytometry. (**A**) (i) Representative flow cytometry histogram result for single cells from breaking RH30 sarcoma spheroids. Cells were incubated with either PBS (red), free IR820 NIR dye (blue), or IR820 NIR dye loaded siRNA linked MSNPs (orange) for 24 h. (ii) Results from the normalized median fluorescence intensity of flow cytometry results for single cells from breaking RH30 sarcoma spheroids. Cells were incubated with either PBS, free IR820 NIR dye, or IR820 NIR dye loaded siRNA linked MSNPs for 24 h. Data are presented as mean ± SD for each individual cell line (*n* = 3 independent experiments of triplicates). (**B**) Results from the normalized median fluorescence intensity of flow cytometry results for single cells from breaking RH30 sarcoma spheroids. Cells were incubated with either free IR820 NIR dye, or IR820 NIR dye loaded siRNA-s-s-MSNPs for different period of times ranging from 0~24 h. The statistical analysis of MFI data was compared between two groups treated with either free dye or dye loaded nanoparticles for the same incubation time (10 min, 1 h, 2 h, 4 h, 8 h, or 24 h). The control group was used to normalize for the inherent fluorescent background of the cells. Data are presented as mean ± SD for each individual cell line (*n* = 3 independent experiments of triplicates). Significance was tested using a two tailed t-test compared to the cells with only buffers for each cell line (ns: not significant, * *p* ≤ 0.05, ** *p* ≤ 0.01).

IR820 NIR dye was loaded into the siRNA linked MSNPs and incubated with RH30 spheroids. The cellular uptake of IR820 was monitored using flow cytometry on cells disintegrated from spheroids after 24 h of incubation. The IR820 loaded siRNA-MSNPs group showed a significant peak shift, indicating a much higher amount of dye per cell compared with incubation with free IR820 dye (Figure 5A(i)). The IR820 content was then quantified with the Median Fluorescence Intensity (MFI) data from flow cytometry. As it was shown in Figure 5A(ii), the MFI only reached 572 ± 39.42 with the free IR820 penetration. However, the IR820 loaded siRNA-MSNPs group showed an over two-fold increase with MFI levels reaching 1194 ± 33.54 after 24 h incubation. 

To further investigate the penetration and uptake speed within RH30 spheroids, time-dependent intracellular MFI was monitored from 10 min to 24 h. It was found that the signal was higher in IR820 loaded siRNA-MSNPs groups after 1 h and 4 h of incubation compared with free IR820 groups. However, this trend was not constant for less than 8 h incubation. It could be noticed that for the 10 min, 2 h and 8 h incubation groups, the difference between the free IR820 group and the IR820 loaded siRNA-MSNPs group was insignificant. Thus, we set up a 24 h group which was considered as long enough for all free IR820 or IR820 loaded siRNA-MSNPs to penetrate to the deepest extent within the spheroid structure. It could be noticed that the MFI of IR820 loaded siRNA-MSNPs incubation was significantly higher than free IR820, with 532 ± 47 compared with 302 ± 20, respectively (Figure 5B).

**Figure 6:**
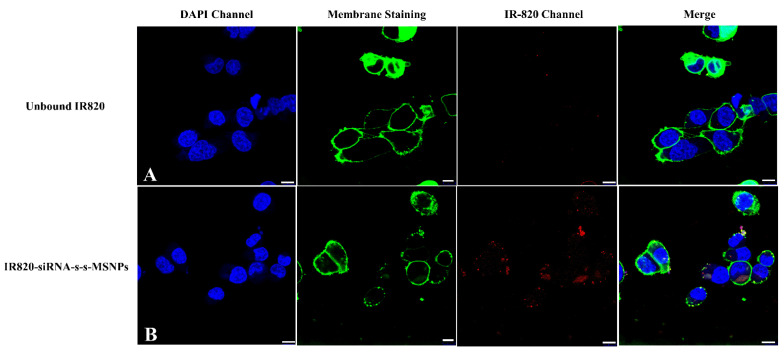
Fluorescence confocal imaging of efficient IR820 cell penetration delivered with MSNPs compared with unbound staining. The RH30 sarcoma cells were incubated overnight with (**A**) unbound IR820; (**B**) IR820 loaded siRNA linked MSNPs. The cells were then thoroughly washed and fixed with 4% PFA. Scale bar, 10 μm.

Cellular localization of siRNA-IR820-MSNPs was investigated using confocal microscopy. CellBrite™ green cytoplasmic membrane staining and DAPI nucleus staining indicated that the IR820 signal was present in the cytoplasm, but outside the nucleus. The sensor of confocal microscopy was not as sensitive as the flow cytometry which required a higher amount of IR820 intracellular to show visible signals. From the images, it could be noticed that the unbound IR820 group did not show any signal after overnight incubation under the confocal microscopy, which indicated relatively low delivery or penetration efficiency (Figure 6A). Nevertheless, the IR820 loaded siRNA-MSNPs group showed significantly visible signals in the IR820 channel under confocal microscopy, which indicated that the MSNPs could deliver a significantly higher amount of IR820 into the cells with a very limited number of nanoparticles applied for incubation (Figure 6B). This results also corresponded to the flow cytometry result. 

**Figure 7:**
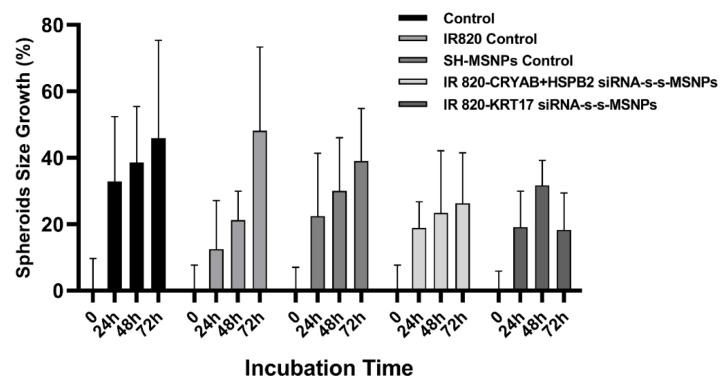
Measurement of IR820 loaded siRNA linked MSNPs effects on cell proliferation. Average spheroid growth in diameter results comparing the relative cell survival rate of RH30 sarcoma cells after treatment with PBS, unbound IR820, SH-MSNPs, IR820-MSNPs linked with CRYAB and HSPB2-targetted siRNA, or IR820-MSNPs linked with KRT17-targetted siRNA, for different periods of time, ranging from 0—72 h (*n* = 6). The relative growth percentage was calculated by comparison between the size of the spheroids in each group before and after the treatment for 0–72 h. Data are presented as mean ± SD for each individual cell line.

Any negative effects of IR820 on cell growth and proliferation, or the functioning of knockdown siRNA, were examined. siRNA-IR820-MSNPs were incubated with RH30 spheroids and compared with PBS controls, free IR820 dye, and SH-MSNPs control. The size of spheroids was monitored at *t* = 0 and 24 h, 48 h and 72 h. After 72 h, spheroids incubated with free IR820 had increased by 48.15% ± 25.22 compared to spheroids in the PBS control, which grew 45.90% ± 29.46. Incubation of the spheroids with SH-MSNPs showed a slightly slower growth rate of 39.01 ± 15.82%, although this was not statistically different from the PBS control. However, incubation with the therapeutic siRNA-IR820-MSNPs showed a clear suppression in the growth rate. The spheroids grew much slower and the growth almost ceased after 48 h; between 48 h and 72 h, spheroids incubated with NPs loaded with CRYAB and HSPB2 only grew 2.89%. Furthermore, the KRT17 siRNA-IR820-MSNPs showed signs of shrinkage and collapse in treated RH30 spheroids after 48 h, with growth dropping back from 31.67 ± 7.53% to 18.29 ± 11.06% after 72 h incubation (Figure 7).

Similar experiments were performed for the RD spheroids with the same incubation groups. The spheroids in all groups grew around 30% in diameter after 72 h, which showed no influence from the siRNA knockout for either CRYAB HSPB2 DKO or KRT17 (Supplementary Figure S5). Thus, showing the specificity of these genes towards aRMS cells. 

### 3.6. Measurement of RMS Intracellular GSH/GSSG Level

**Figure 8:**
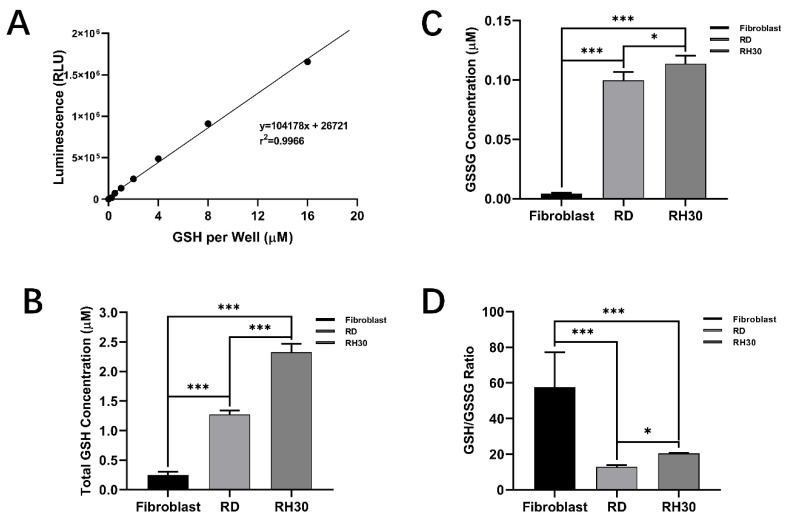
Quantification of intracellular GSG and GSSH concentration and GSH/GSSG ratio. (**A**) Standard curve plotted from eight standards (concentration ranging from 0.25 to 16 μM) which was treated under the same condition as cell samples. (**B**) Quantification of total GSH level of fibroblasts, RD and RH30 cells under the same conditions. (**C**) Quantification of GSSG level of fibroblasts, RD and RH30 cells under the same conditions. (**D**) GSH/GSSG ratio of fibroblasts, RD and RH30 cells under the same conditions (*n* = 6). Data are presented as mean ± SD for each individual cell line. Spheroids growth results comparing the cell relative survival rate and metabolism level of RH30 sarcoma cells after treatment with PBS, unbound IR820, SH-MSNPs, IR820 loaded CRYAB and HSPB2-target siRNA linked MSNPs, or IR820 loaded KRT17-target siRNA linked MSNPs for different periods of times ranging, from 0~72 h (*n* = 6). Data are presented as mean ± SD for each individual cell line. Significance was tested using a two tailed t-test compared to the fibroblasts for each cell line (ns: not significant, * *p* ≤ 0.05, *** *p* ≤ 0.001).

Experiments were performed to validate that RMS cells, especially the targeted aRMS (RH30) cells, have significantly higher levels of GSH than normal cells. A luminescence method was used for the assessment. Eight standards of known concentrations of GSH were used to generate a standard curve (Figure 8A). All three cell lines were seen to have a total GSH level in the micromolar range: fibroblasts 0.25 ± 0.06 μM, RD cells 1.27 ± 0.07 μM, and RH30 cells 2.33 ± 0.14 μM (Figure 8B). Compared with the fibroblasts, the two RMS cells lines both showed a several-fold increase for the intracellular total GSH amount, i.e., 5.08 and 9.28 times higher for RD and RH30 cells, respectively. 

The GSSG level was also measured at the same time. As most of the intracellular GSH was normally kept as the reduced form, the GSSG levels were all relatively low; fibroblasts 0.0045 ± 0.0005 μM, RD cells 0.0997 ± 0.0071 μM, and RH30 cells 0.1137 ± 0.0067 μM (Figure 8C). The ratios showed a different trend as the fibroblasts had the highest GSH/GSSG ratio: fibroblasts 57.59 ± 19.60, RD cells 12.83 ± 1.04, and RH30 cells 20.46 ± 0.32. Although there was a significant difference between RD and RH30 cell groups, the GSH/GSSG ratio decreased more than two-fold for both of the groups compared with the normal fibroblast cells (Figure 8D). 

## 4. Discussion

### 4.1. The Selected siRNAs could Efficiently Suppress the aRMS Growth

Previous studies in other cancer cell lines have shown that the CRYAB and HSPB2 double knockout (DKO) can play an important role in inducing cell apoptosis and necrosis [29]. Moreover, it has been shown that the CRYAB and HSPB2 gene expression level was related to the human renal carcinogenesis through direct regulation of cell ROS levels and the Warburg effect [29]. HSPB2 has specific interactions with the outer mitochondrial membrane, which directly affects mitochondrial functionality after CRYAB and HSPB2 DKO by changing the mitochondrial membrane permeability [30]. Alterations to the mitochondrial permeability transition and subsequent Ca^2+^ overload result in the CRYAB and HSPB2 DKO, directly leading to necrotic and apoptotic cell death [30]. Herein it has been shown that the reduction of RH30 cell viability and mobility was mainly due to apoptosis and necrosis, i.e., a 50% reduction of the population after 72 h of transfection (Figure 4B), which further indicated that mitochondrial permeability could be the main cause of siRNA gene knockdown-related viability suppression. 

Expression level studies have previously shown that both CRYAB and HSPB2 are abnormally upregulated in aRMS compared with eRMS. In microarray evaluation of gene expression, CRYAB and HSPB2 were seen to be upregulated 13.52-fold and 12-fold in RH30 and RD cells, respectively [18]. In fact, these genes showed two of the most significantly upregulated expression levels [18].

Therefore, it has been further validated in our study that both viability (Figure 1A(ii) and mobility (Figure 1B(ii)) of aRMS cells was significantly suppressed after CRYAB and HSPB2 DKO transfected with siRNA concentrations as low as 12.5 nM after 24 h. Only small differences were seen in the eRMS groups due to the significantly lower initial level of expression for both genes. 

Similarly, upregulation of KRT17 has been used as a diagnostic marker for several cancers, such as breast cancer [31], ovarian cancer [32] and skin squamous carcinoma [33,34]. Knockdown of KRT17 using targeting siRNA has been shown to significantly suppress gastric cancer cell proliferation (42.36 ± 3.2%) and migration (37.2 ± 6.2%) through the AKT/mTOR pathway [34]. The KRT17 gene has also been shown to be critical for proliferation of osteosarcoma [35], and for cellular adhesion and oncogenic transformation in Ewing’s sarcoma [36]. Knockdown of KRT17 may also decrease the viability and Warburg effect of osteosarcoma cells by significantly affecting the AKT/mTOR/hypoxia-inducible factor 1α (HIF1α) pathway and interrupting the expression of relevant genes [35]. However, the effects of knockdown of KRT17 in aRMS cells has not been previously demonstrated. The experimental results demonstrated that the knockdown of KRT17 could efficiently decrease the cell viability (Figure 1A(ii)) and cell mobility of aRMS cells, which may result from AKT/mTOR pathway which was confirmed in other types of sarcomas (Figure 1B) [35]. However, it should be noted that in eRMS cells, growth was significantly suppressed, with KRT17 siRNA concentrations as low as 5 nM after 24 h. This indicates that eRMS cells are very sensitive to KRT17 knockdown even with a significantly lower initial expression level compared with aRMS cells (Figure 4A(i)) [18]. Moreover, after a certain concentration threshold, the decrease in aRMS cell viability does not show a linear relationship with the concentration of the siRNA applied for gene knockout (Figure 1A). Similar results are seen in the literature in other cell lines [35]

### 4.2. MSNPs can Deliver the Targeting siRNA Safely and Effectively 

This study aimed to synthesize a nanoparticle to deliver siRNA in a controlled and targeted manner. DharmaFect™ is a frequently used commercial RNA transfection reagent based on a cationic lipid. However, it results in cellular toxicity [37,38], is unstable, expensive, and cannot be directly applied in vivo [37] In contrast, MSNPs have excellent stability and biocompatibility, and are promising candidates for siRNA delivery [39,40,41] In our study, thiol functionalized siRNA was bound to SH-MSNPs through the formation of disulfide bonds. The disulfide bonds ensured stable and reliable binding before controlled release of the siRNA [42]. The stability of the complex was validated using gel electrophoresis with no visible leakiness (Figure 3A).

In vivo, RNase is an obstacle for siRNA-based gene therapy [6]. There have been multiple different routes to solve this issue, for example, 2′-*O*-methyl (2′-OMe) and 2′-deoxy-2′-fluoro (2′-*F*) modifications to siRNA have shown better in vitro potency and stability [43], and numerous lipid-based carriers for siRNA delivery have been trialled, including the commercially approved Patisiran (ONPATTRO™) [44]. The siRNA in our study was 21 bp and could therefore be easily degraded completely within 15 min (Figure 3B(i)), which was in agreement with the short half-life of 6 min for siRNA clearance seen in other studies. MSNPs showed strong protective functionality of the carried siRNA, as also seen in other publications [45]. The protection by MSNPs was mainly due to (1) Electrostatic repulsion: the MSNPs were negatively charged (Supplementary Figure S2), and the siRNA linked by the disulfide bonds increased the negative surface charge, which led to a strong repulsive effect on RNase I, which itself has been proven to have an initial negative charge [46], and (2) Steric protection: the silica nanoparticle core was mesoporous and so a large proportion of the siRNAs were linked and contained within small pores. The RNase I, which is a large molecule, was unable to access and degrade the siRNAs. 

Compared with the DharmaFect transfection group, the siRNA-MSNP group showed similar or greater knockdown efficacy evidenced by significant apoptosis and necrosis to the cell population (Figure 4). 

### 4.3. GSH within aRMS Cells could Be Applied for Targeted Release of siRNA

Several studies have indicated that GSH could be used as an intracellular stimuli response factor for active release of a specific drug into cells, especially in cancer cells. Yang et al. reported a cancer cell specific degradable dendritic mesoporous organosilica nanoparticle (DDMON) which used the differences in GSH concentration for selective release. The release mechanism was based on the disulfide bond (-S-S-) incorporation in the DDMONs. The high concentration of GSH in the cytoplasm of cancer cells would lead to the disintegration of the NP structure by the reduction of the disulphide bonds, and the release of encapsulated therapeutic agents. The DDMONs that were generated carried the cancer therapeutic PEI/ RNAse (Polyethylenimine/Ribonuclease A) A-Aco-FITC (cis-aconitic acid-Fluorescein isothiocyanate) complex. Incubation of the complex in melanoma (B16F0) cell lines resulted in significantly higher cell kill compared to the same amount of the loaded non-biodegradable dendritic mesoporous organosilica nanoparticles (DMONs). As a control the constructs were tested in normal HEK293t cells, and viability was higher after treatment with DDMONs than DMONs. This confirmed the selectivity of the system based on the GSH concentration, thereby providing protection for healthy cells [17,47]. The GSH levels we found in aRMS cells were nearly 10 times higher than normal fibroblast cells (Figure 8B). This further indicated that the GSH within RH30 cells could be applied for targeted release of nanoparticle associated siRNA. 

Moreover, within Figure 4A(ii), it should be noted that all siRNA transfection groups, whether transfected using DharmaFect or MSNPs, show significantly decreased cell viabilities after 24 h. However, knockdown with 5 nM KRT17 siRNA (Figure 4A(i)) using DharmaFect showed significant suppression of cell growth which was not seen with SH-MSNPs. Examination of the GSH data from Section 3.6 explains this outcome, since the GSH levels were insufficient to release the siRNA from the nanoparticles. Hence, the GSH/GSSG level was crucial for the system to function.

### 4.4. MSNPs can Enhance the Penetration of the Loaded IR820 Dye into 3D Spheroids

Our system combined the IR820 NIR signalling with MSNPs-induced siRNA knockdown. To our knowledge, there have been no studies similar to our system for the RMS theranostic. Previous studies have shown that IR820 could be effectively loaded into MSNPs with a relatively good capacity of 10.0  ±  0.8%, with the hydrophobic–hydrophobic interaction supporting its amphiphilic functionality [48]. Furthermore, it has also been discovered that the MSNPs could enhance the stability and energy conversion efficiency of loaded IR820 [48,49]. In our study, the amount of delivered IR820 was compared between the free IR820 and loaded IR820. Different from the free dye which normally relied on passive diffusion, the endocytosis of MSNPs could greatly improve the uptake of loaded IR820 [13], especially within the 3D spheroids with much more complex structures, which had different concentrations from outside to inside and mainly relied on the penetration ability (Figure 5B). It was validated by the flow cytometry results which showed a clear peak shift and over a two-fold increase in MFI (Figure 5A). It was further confirmed with the confocal images which showed signals, with a clear and stable IR820 signal for the IR820 loaded siRNA-MSNPs group. The uptaken IR820 was kept within the endosomes. On the contrary, the free IR820 group showed no signal at all due to low uptake and diffusion out of the cells after the fixation (Figure 6). For the time-dependent uptake evaluation with RH30 sarcoma spheroids, the trend between free IR820 and loaded IR820 was not clear for the first few hours. However, the ultimate significant MFI difference after 24 h demonstrated the deeper penetration depth assisted by the MSNPs. 

Moreover, IR820 dye could effectively enable the non-invasive biomedical sensing due to decreased tissue scattering, better imaging resolution and deeper penetration depth [50,51]. Decreased tissue scattering could also prevent the issue of low signal-to-noise ratio due to the high tissue background noise [51]. The addition of an NIR dye to the nanoparticle would enable tracking both in vitro and in vivo through penetration across the skin at centimetre depth, within a transparency window so that blood does not interfere with the signal [25].

## 5. Conclusions

In conclusion, after successful validation of the knockdown of selected targets (CRYAB and HSPB2, and KRT17) for aRMS suppression, the targeting siRNA was successfully linked to the surface of characterized SH-MSNPs with disulfide bonds. The MSNPs were proved to successfully deliver, transfect, and protect the siRNA. Compared with the transfection group using commercially available DharmaFect, all siRNAs delivered and transfected by the designed nanosystem were found to have a similar or better transfection efficiency, with significant suppression of viability and mobility within both 2D and 3D RH30 cell models. On the contrary, no change of viability or mobility was observed for the eRMS RD cell spheroid model after the transfection. The siRNA was protected from RNase I degradation for over 24 h, compared with free siRNA which was degraded within a few minutes. Subsequently, IR820 dye was loaded into the siRNA linked MSNPs to allow imaging of the nanoconstruct in vivo. The GSH and GSSG levels were precisely quantified for fibroblasts—RD and RH30 cells that indicated that the GSH levels within RH30 cells were sufficient for targeted release from the nanoparticle core. The successful building of the theranostic nanosystem, which combines the targeting siRNAs therapy and IR820 NIR monitoring, could provide another route for tackling the aRMS in a more efficient and non-invasive way. In addition, our previous study identified aRMS specific aptamers that could be conjugated onto the current nanoconstruct to further enhance the specificity and efficiency of targeted siRNA delivery and therapy [52].

## Data Availability

Data supporting reported results can be found at Mendeley Data, V1, doi: 10.17632/fprbxjtrzy.1.

## Supplementary Materials

The following supporting information can be downloaded at: https://www.mdpi.com/article/10.3390/biom12081129/s1, Figure S1: Zeta Size Distribution by number of Synthesized SH-MSNPs; Figure S2: Zeta Potential of Synthesized SH-MSNPs (Neutral pH); Figure S3: Illustration of scratch assay measurement and equation; Figure S4: Measurement of RD spheroids treated for 72 h with PBS, unbound IR820, SH-MSNPs, IR820-MSNPs linked to CRYAB & HSPB2-targetted siRNA, or IR820-MSNPs linked to KRT17-targetted siRNA (*n* = 6); Figure S5: Diameter of RD spheroids after treatment for 72 h with PBS, unbound IR820, SH-MSNPs, IR820- MSNPs linked to CRYAB & HSPB2-target siRNA, or IR820-MSNPs linked to KRT17-target siRNA (*n* = 6).
