# Supplementary material for: Exploitation of High Tumour GSH Levels for Targeted siRNA Delivery in Rhabdomyosarcoma Cells
Source: Biomolecules. 2022 Aug 17;12(8):1129. doi: 10.3390/biom12081129 (PMC9405954; doi:10.3390/biom12081129)
Supplement: Supplementary file 1 [file biomolecules-12-01129-s001.zip › biomolecules-1857529-supplementary.pdf]

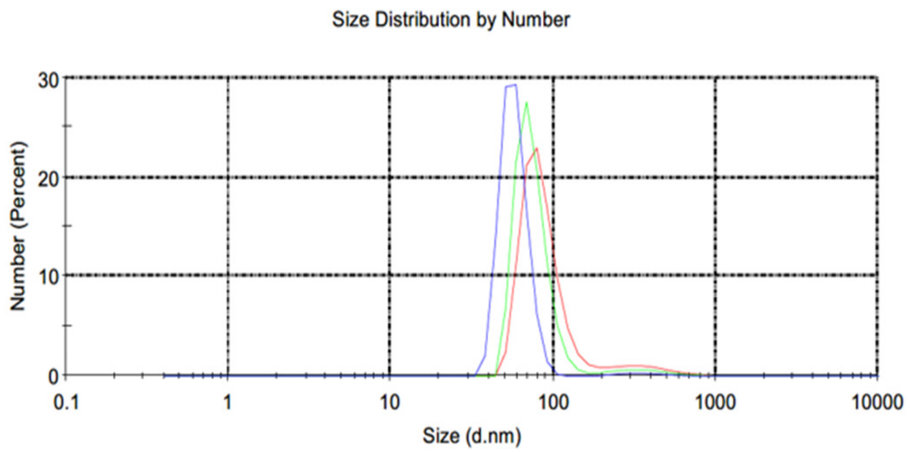

Supplementary Figure S1. Zeta Size Distribution by number of Synthesized SH-MSNPs.

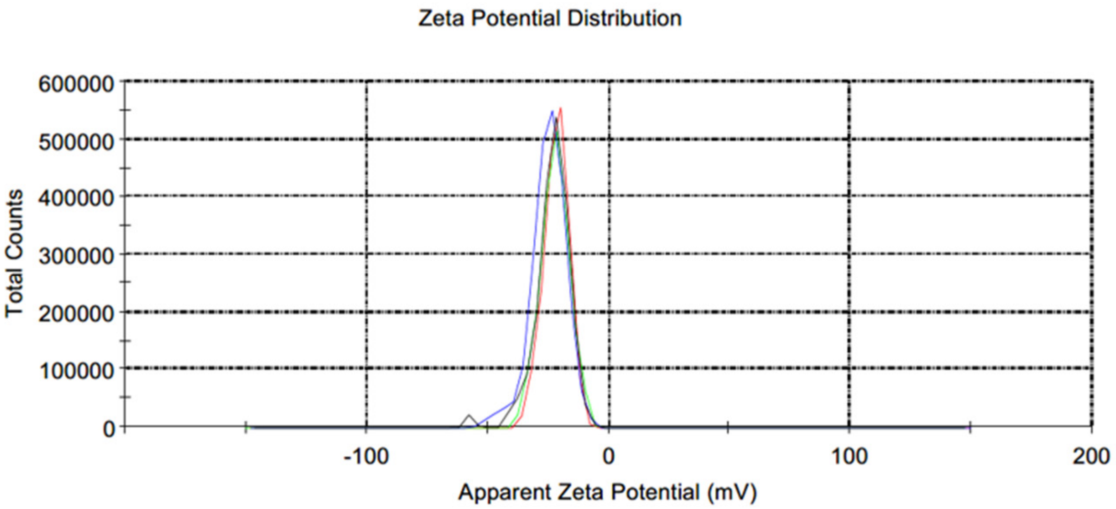

Supplementary Figure S2. Zeta Potential of Synthesized SH-MSNPs (Neutral pH).

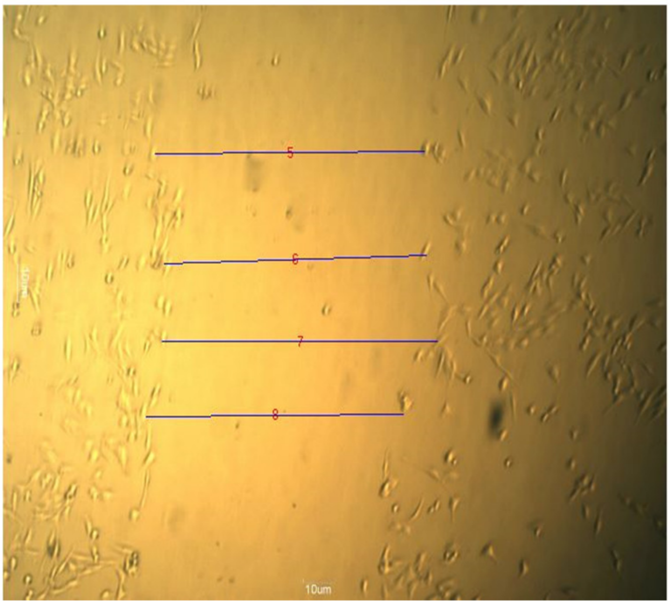

$$\frac{W_{before} - W_{after}}{W_{before}} \times 100\ \%$$

Supplementary Figure S3. Illustration of scratch assay measurement and equation.

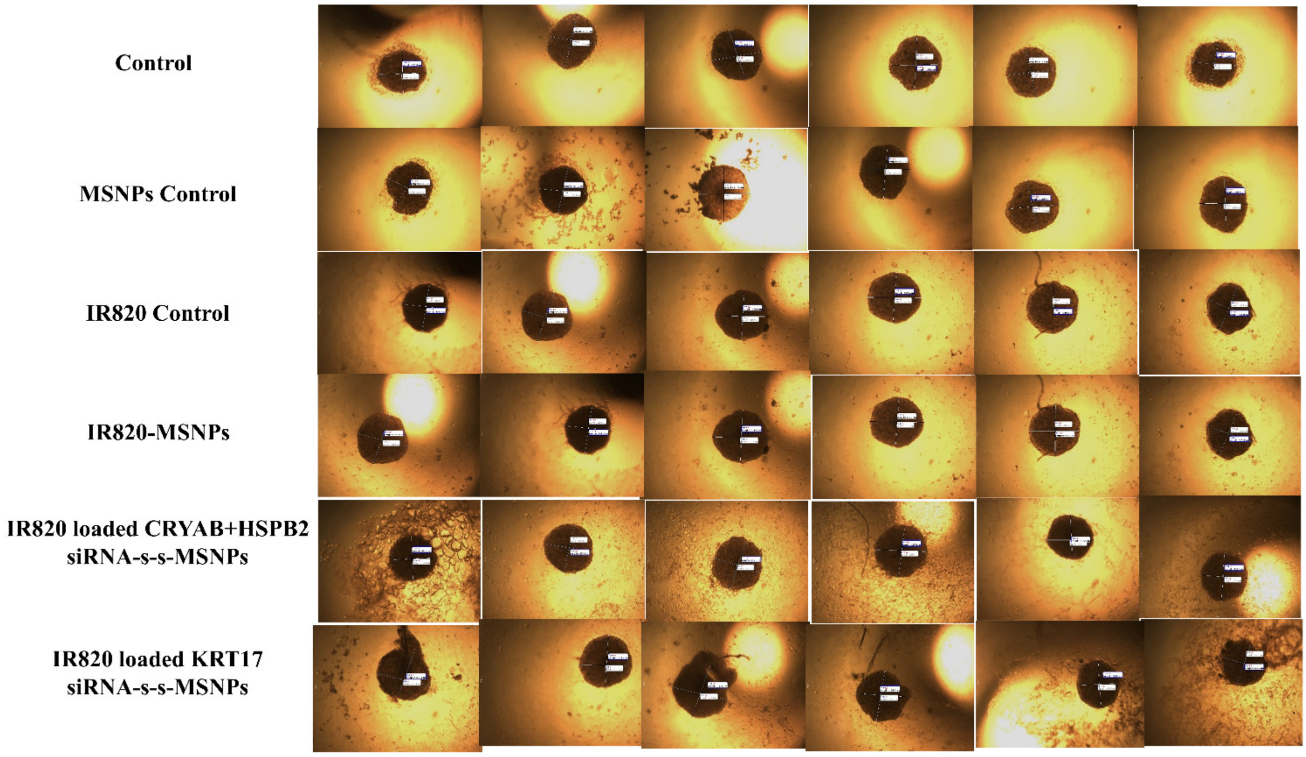

Supplementary Figure S4. Measurement of RD spheroids treated for 72 hours with PBS, unbound IR820, SH-MSNPs, IR820-MSNPs linked to CRYAB & HSPB2-targetted siRNA, or IR820-MSNPs linked to KRT17-targetted siRNA (n = 6).

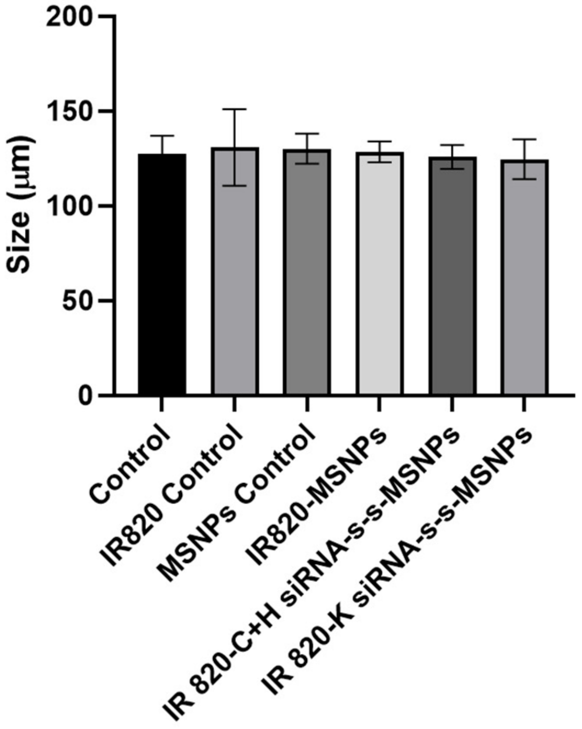

Supplementary Figure S5. Diameter of RD spheroids after treatment for 72 hours with PBS, unbound IR820, SH-MSNPs, IR820-MSNPs linked to CRYAB & HSPB2-target siRNA, or IR820-MSNPs linked to KRT17-target siRNA (n = 6).
